# Analysis of the Influence of Psychological Contract on Employee Safety Behaviors against COVID-19

**DOI:** 10.3390/ijerph17186747

**Published:** 2020-09-16

**Authors:** Yuexin Du, Hui Liu

**Affiliations:** 1 Department of Human Resource Management, School of Business Administration, Zhejiang University of Finance & Economics, Hangzhou 310018, Zhejiang, China; duyuexin@zufe.edu.cn; 2 Institute of Human Resource Management, Zhejiang University of Finance & Economics, Hangzhou 310018, Zhejiang, China

**Keywords:** COVID-19, psychological contract, safety behavior, job burnout, perceived insider status, double mediators

## Abstract

This study explored the influencing factors of safety behavior from the perspective of employees, studied the mechanism of the psychological contract on employees’ safety behavior in the context of the Chinese epidemic situation, tested the mediating role of job burnout and perceived insider status in the process of work resumption, and provided preventive suggestions for combating the global spread of COVID-19. A questionnaire survey was utilized to collect data and, combined with the necessary protective measures taken for employees in China, was used to modify the mature safety behavior scale. Finally, through the analysis of 402 employees’ questionnaires, the hypotheses were verified; that is, in the process of Chinese enterprises returning to work to cope with COVID-19, the psychological contract has a positive role in promoting employees’ safety behavior, while job burnout plays a weakened mediating role, and perceived insider status plays a strengthening mediating role. The psychological contract negatively affects job burnout but positively affects perceived insider status. Job burnout negatively affects employees’ safety behavior, but perceived insider status positively affects employees’ safety behavior. The results show that employees’ conscious participation in safety behavior plays an irreplaceable role in the prevention of COVID-19 and safety of work resumption.

## 1. Introduction

In mid-December 2019, the outbreak of COVID-19 (coronavirus disease 2019) in Wuhan, Hubei Province in China has attracted global attention. With the passage of time, it quickly spread to all parts of China and other countries [1] and greatly threatened the safety of people’s production and life [2] and even led to the shutdown of a large number of enterprises in many countries. The COVID-19 crisis represents a new type and quality of challenge for companies [3]. However, while dealing with the epidemic situation, people must also ensure the smooth operation of the economy. Therefore, it is imperative for enterprises to resume work in the affected countries; some enterprises had to recall employees to restart production activities. It is very important to eliminate employees’ fear of COVID-19 and take effective protective measures [4]. China has taken the lead in controlling the spread of COVID-19 in the short term [5], and the overall situation of prevention is stable [6]. It is very beneficial to study the safety behavior practice of its enterprises and employees from the perspective of organizational psychology, which is of great significance for ensuring occupational health and improving the ability of enterprises to cope with COVID-19 and other public health crises.

With the deepening of the research on the influence of psychological factors on employees’ safety behavior, mature concepts and theories in organizational psychology are gradually being applied to research in the field of safety, such as leadership style, organizational climate, organizational support, etc. [7]. With the aggravation of the global epidemic situation to fight the spread of COVID-19 and the arduous task of work resumption, enterprises will face many health problems. The psychological contract, as an implicit contract between employees and organizations, plays an important role in regulating employees’ work behaviors [8]. Many studies show that there is a significant positive correlation between the psychological contract and employees’ work behavior, and this has an impact on the employee’s work behavior and performance through mediating variables [9,10]. Among them, as a resulting variable of the psychological contract [11,12], employees’ job burnout will have a negative impact on employees’ safety behavior, while employees with higher perception of internal identity will show more positive behaviors in their work [13,14].

At present, research on the independent concept of the psychological contract is already abundant. However, there is little research on other variables as mediators to study the psychological contract. For example, there is little research on the relationship between the psychological contract and employees’ safety behavior by using perceived insider status as intermediary variable. In addition, the research on the relationship between the psychological contract and employees’ safety behavior mainly focuses on high-risk areas such as construction and mining, while the research on the public health crisis is less. However, due to the influence of COVID-19, employees’ safety behavior is directly related to health problems and customer safety, and it is necessary to explore whether the psychological contract will affect the safety behavior of employees in work resumption in many industries to fight the spread of COVID-19. In view of this, this paper selected Chinese employees as the research object, combined with job burnout and perceived insider status, to explore the impact of the psychological contract on employees’ safety behavior. We verified the mediating role of job burnout and perceived insider status between the psychological contract and safety behavior and revealed the formation mechanism of enterprise employees’ safety behavior in the epidemic environment, hoping to make up for the deficiency of existing research in theory. At the same time, China as a country with rapid resumption of work in this epidemic situation has made certain achievements; for example, many enterprises began to resume production in late February—except for high-risk areas such as Wuhan, enterprises in various regions basically resumed operation in March. This shows that the research on Chinese enterprises’ response to COVID-19 can provide useful enlightenment for enterprises in the world to stabilize employees’ emotions, manage employees’ safety behaviors and deal with such crises in the future.

## 2. Literature Review

### 2.1. Psychological Contract and Safety Behavior

The psychological contract is the sum of implicit and unexplained mutual expectations between organizations and employees [15]. This expectation, on the one hand, reflects the individual interests of the organization members; on the other hand, it also reflects the interest concession made by the organization leaders who realize the collective interests. When the organization reaches a consensus on the expectation of the members in an unspecified way, the psychological contract is achieved [16]. Relevant theories point out that the generalized psychological contract emphasizes the subjective feelings and cognition of the organization and employees for each other’s responsibilities and obligations from the perspectives of the organization and employee [17]. However, the narrow definition of the psychological contract is only from the perspective of employees, emphasizing that the psychological contract reflects employees’ feelings and cognition of each other’s responsibilities in the exchange relationship [18]. The theoretical basis of the psychological contract is equity theory and social exchange theory [19]. The former proposes that, in the employment relationship between the organization and employees, employees have the obligation to perform their duties and have the responsibility to identify with the organization in exchange for the emotional and economic returns of the organization, such as opportunities for learning and promotion and a good working environment. The latter, combined with affective events theory, believes that, when such a fair economic exchange or the perceptional balance of emotional exchange is broken, the level of organizational identity and job satisfaction will be reduced, and the risk of psychological or behavioral counterattack will be increased.

As an outcome variable of the psychological contract, employees’ safety behavior has always been the focus of attention in the development of enterprises. Safety behavior is defined as the behavior through which people abide by the operating rules at work, as well as all behaviors made for the purpose of ensuring their own safety and ensuring the integrity of equipment and other materials in the process of accidents [20]; it may also refer to a series of conscious behaviors made by people in the process of work to avoid accidents affecting their own safety [21]. Neal has divided the dimensions of safety behavior, which consists of safety obedience behavior and safety participation behavior [22]. Among them, safety obedience behavior is to work in a safe way, according to the safety procedures and steps that are stipulated by the organization. Safety participation behavior is to actively participate in the safety work and to complete the safety work beyond the normal organization regulations, which have the characteristics of initiative and voluntariness.

Relevant research shows that, if the communication between the organization and employees is not smooth or the employees feel that the enterprise has not fulfilled the corresponding obligations or responsibilities, the psychological contract will be violated or broken, which will lead to a negative emotional experience [23], and this experience will lead to a negative impact on the work behavior of employees. The study found that the breach or violation of the psychological contract has a significant impact on task performance and the organizational performance of employees [24]. Based on the perspective of the psychological contract, enhancing the sense of safety responsibility and avoiding the negative emotions of employees can enhance their sense of belonging and cohesion, make them understand the enterprise more and reach an agreement with the enterprise in terms of safety protection, so as to improve their safety behaviors [25] and strengthen the prevention of COVID-19. Therefore, this study proposed the following hypotheses:

**Hypothesis** **1** **(H1).** *Psychological contract has a positive predictive effect on the safety behavior of employees to prevent the spread of COVID-19.*

### 2.2. The Mediating Role of Job Burnout

Job burnout refers to a kind of negative working state of employees under a continuous and intense working environment. This kind of working state is manifested in three aspects: emotional exhaustion, depersonalization and low personal accomplishment [26]. Among them, emotional exhaustion refers to the excessive consumption of individual emotional resources, which represents the pressure dimension; depersonalization refers to the negative, indifferent and excessively alienated attitude towards the service object, which represents the interpersonal dimension; low personal accomplishment refers to the denial of the value and competence of one’s own work, which represents the dimension of self-evaluation. Siegrist proposed the “effort–reward model” of job burnout from the perspective of social exchange theory [27]. The model believes that, if the individual cannot effectively solve the situation of unequal effort and return for a long time, tension or anxiety will accompany the individual’s development and carries the risk of leading to job burnout. From the perspective of resource preservation theory, social competence theory and other theoretical perspectives, relevant research has also explored the formation mechanism of job burnout and confirmed that the formation of job burnout has a certain relationship with employees’ own state and work environment [28].

Relevant studies have pointed out that the psychological contract directly determines what kind of work attitude and behavior of employees will take [29]; the individual psychological contract is significantly related to work engagement, and the violation of the psychological contract can predict job burnout and its three dimensions [30]. Resource conservation theory [31] also pointed out that, when individuals face diverse pressures in the organization, they need to mobilize all kinds of resources to cope with them. If the continuous physical and psychological efforts are not supplemented in a timely manner, the physical and mental resources will be consumed and exhausted, which will affect the individual’s ability to deal with the danger. However, empirical research showed that the violation of the psychological contract will cause employees have a series of negative emotions such as job burnout [29], and job burnout has a direct effect on safety behavior [32]. Job burnout will induce psychological diseases [33], thus affecting safety operating procedures and production behavior, which is not conducive to the prevention of COVID-19 in work resumption. Therefore, this study proposed the following hypotheses:

**Hypothesis** **2** **(H2).** *Job burnout plays a mediating role in the influence of the psychological contract on employees’ safety behaviors to prevent the spread of COVID-19.*

**Hypothesis** **3** **(H3).** *Psychological contract has a negative predictive effect on employees’ job burnout in preventing the spread of COVID-19.*

**Hypothesis** **4** **(H4).** *Job burnout has a negative predictive effect on employees’ safety behaviors to prevent the spread of COVID-19.*

### 2.3. The Mediating Role of Perceived Insider Status

Perceived insider status originated from the division of “insiders” and “outsiders”; it is used to study the relationship between organizations and employees in the field of organizational behavior. Specifically, it refers to the value that employees feel and the degree to which they are treated as “insiders” by the organization. It is a kind of cognition of employees in their employment relationship [34] and emphasizes employees’ sense of belonging within the organization [35]. In recent years, the perceived insider status has become a relatively new angle from which to study the relationship between organizations and employees. From the perspective of motivation-hygiene theory, employees’ perceptions of internal identity mainly come from two aspects: one is self-reinforcing, which belongs to internal causes; the second is organizational giving and feedback, which is external stimulation [36]. In terms of function, perceived insider status is one of the criteria used to measure whether a member is an insider of an organization [34], and this perception will further affect the individual’s work emotions and conscientiousness. When employees feel that they are “insiders”, they will increase their sense of organizational identity and positively evaluate the resources and services provided by the organization [37].

Relevant scholars have explored the relationship between psychological contract and perceived insider status, and the results of this research show that the psychological contract also has a significant positive effect on employees’ perceptions of internal identity [38]. From the perspective of social exchange theory, the more internal identity perception that employees experience, the more organizational citizenship behavior they will show, which will lead to less deviation and unsafe behavior at work [39]. Stamper and Masterson also believe that the higher the level of employees’ perception of internal identity, the more likely they are to have altruistic behavior and reduce production deviant behavior [34]; this is conducive to the prevention of COVID-19. Therefore, this study proposed the following hypotheses:

**Hypothesis** **5** **(H5).** *Perceived insider status plays a mediating role in the influence of the psychological contract on employees’ safety behavior to prevent the spread of COVID-19.*

**Hypothesis** **6** **(H6).** *Psychological contract has a positive predictive effect on employees’ perceived insider status in preventing the spread of COVID-19.*

**Hypothesis** **7** **(H7).** *Perceived insider status has a positive predictive effect on employees’ safety behaviors to prevent the spread of COVID-19.*

**Figure 1:**
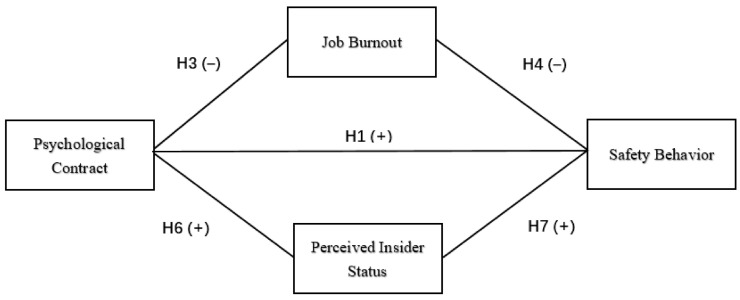
Conceptual framework.

Based on the above hypotheses, this study presented the following conceptual framework, as shown in Figure 1.

## 3. Materials and Methodology

In this study, a questionnaire survey was utilized to collect data, and combined with the research content, corrected and adjusted the unclear items and finally formed the items of the questionnaire. The reliability and validity of the scale were tested and exploratory factor analysis was utilized to ensure the quality of the scale [40]. Likert’s five-point scoring system was used in all scales, from 1 to 5, indicating “very disagree” to “very agreeable”.

### 3.1. Measures

Measurement of psychological contract: this study adopted the method of three-dimensional division: transaction, relationship and development. Based on the psychological contract scale designed by Chinese scholars Zhang Aiwu and Li Xiyuan, the reliability of each dimension of the original scale was above 0.8 [41]. Combined with the characteristics of this study, this psychological contract measurement scale is designed with a total of 13 questions, and all items were scored positively; for example, “The company provides stable job security for employees”.

Measurement of job burnout: the job burnout scale (MBI-GS) developed by Li Chaoping, a Chinese scholar, has been proven to have high reliability and validity. The reliability of emotional exhaustion, depersonalization and low personal accomplishment in the original scale was 0.88, 0.83 and 0.82, respectively [42]. Considering the research background of this paper, the MBI-GS was selected. There are 15 items in the whole scale, and 9 items adopt positive scoring, such as “I believe that I can effectively complete the work”, while the other 6 items adopt the reverse scoring method, such as “Too much work makes me feel stressed”.

Measurement of perceived insider status: in the existing literature, perceived insider status is regarded as a one-dimensional construct [43]. The representative achievement is a one-dimensional scale with six items that was developed by Stamper and Masterson; the reliability of the original scale is 0.743 [31]. In this study, the scale was adopted, and “I’m an outsider in the company” is one of the items.

Measurement of safety behavior: based on the scale developed by Neal and Griffin, the internal consistency coefficient of this scale is greater than 0.8 [22], and combined with the measures taken by Chinese corporate and employees in response to COVID-19, the safety behavior scale was innovated. It is divided into two dimensions of safety compliance behavior and safety participation behavior, with a total of 9 items. All items were scored in a positive way, and the specific items are shown in Table 1.

### 3.2. Sample and Data Collection

The sample includes manufacturing industry and service industries in 21 provinces and regions in China. The questionnaire survey adopted the form of a combination of online and offline, so as to collect data from different provinces and different industries more widely. In June 2020, a total of 450 questionnaires were distributed; 48 questionnaires with inferior quality such as incomplete or similar answers were excluded, and 402 valid questionnaires were finally obtained. The effective recovery rate of questionnaires was 89.33%. Among them, 125 questionnaires were collected online and 277 questionnaires were collected offline. As the prevention of enterprises in all regions of China tended to be normalized in June, the questionnaire of the 21 regions is very representative of the prevention situation in China.

Demographic variables are considered very important in social science research and these were measured to check the significant direct effect of these control variables on preventive behavior among people about COVID-19 [44]. Essential statistical components are age, gender, education and working years in this paper. The details of these variables are shown in Table 2.

The basic information of the enterprises in which the sample belongs is described and counted: (1) as for the nature of the enterprise, the majority of the subjects are employees of private enterprises, accounting for 73.9%; (2) from the perspective of industry type, 73.9% of the samples were originally from the manufacturing industry, such as clothing manufacturing, food manufacturing, while other samples were mainly from the service industries, including hotels and shopping malls; (3) from the perspective of the time of work resumption in 2020, 86.3% of the employees in the sample returned to work before April. Among them, 279 subjects returned to work in February, accounting for 69.4% of all subjects. This showed that most regions of China resumed work earlier, and the epidemic in Wuhan caused by COVID-19 has not seriously affected the operation of enterprises in other regions of China.

## 4. Data Analysis and Results

In this study, Statistical Product and Service Solutions (SPSS version 25.0 Armonk, NY, USA) was developed by three students of Stanford University in 1968 and this software belongs to International Business Machines Corporation (IBM) company.The latest version 25 was used for data analysis, and the reliability and validity of variables were tested. Through correlation analysis, multiple linear regression analysis and intermediary effect test, the hypothesis test was carried out.

### 4.1. Reliability and Validity Analysis

The reliability of the questionnaire is mainly used to test the internal consistency of the measurement results. This paper uses Cronbach’s Alpha coefficient (α value), which can accurately reflect the internal structure of the scale and consistency of the measurement items [40]. Validity analysis is utilized to determine the validity of the questionnaire and to investigate whether the results of the questionnaire are consistent with the actual situation of the sample [40].

According to the reliability test, α values of psychological contract, job burnout, perceived insider status and safety behavior in this research were 0.910, 0.875, 0.902 and 0.922, respectively, which were higher than the standard of 0.7 and were all greater than 0.8, which indicated that the four scales had very high reliability. In addition, the α value of the overall reliability test reached 0.777; that is, the measurement results had very high consistency, stability and reliability, and the scale passed the reliability testing.

According to the validity test, the results of the KMO value of psychological contract, job burnout, perceived insider status and safety behavior were 0.931, 0.899, 0.862 and 0.915, and the results of Bartlett’s spherical test were also significant at the 0.01 level. The conclusion indicates that these four scales are highly suitable for factor analysis [45]. In this paper, we extracted the sample data and rotated the factors using the maximum variance method to obtain the factor load matrix of each scale after rotation. In the process of factor analysis, we found that there are three factors in the psychological contract, three factors in the job burnout, only one factor in the perceived insider status, two factors in the safety behavior and the structures of them are clear. In addition, the load of each factor is >0.5, and the cumulative variance interpretation rate is 64.027%, 56.690%, 67.384% and 71.145%, which also verifies that they are in line with the dimensions of each scale.

Since the safety behavior scale has been modified and innovated based on the preventive measures against COVID-19 in China, the specific results of factor analysis are shown in Table 3.

According to Table 3, factor 1 is the obedience dimension of safety behavior, including Q1, Q2, Q3, Q4 and Q5, and factor 2 is the participation dimension of safety behavior, including Q6, Q7, Q8 and Q9.

In order to ensure the validity of the modified scale, confirmatory factor analysis was carried out on the four scales. Finally, 11 items of psychological contract, 14 items of job burnout, 5 items of perceived insider status and 9 items of COVID-19 safety behavior were obtained by deleting the items with unsatisfactory standard load coefficients. The results of confirmatory factor analysis can be seen in Table 4.

According to the data in the table above, important indicators of scales all meet the criterion for judgement, indicating that the overall validity of the four scales is better.

### 4.2. Correlation Analysis

Correlation analysis is used to analyze the closeness of the linear relationship between two variables [46,47]. From Table 5, there is a significant correlation between psychological contract, job burnout, perceived insider status and safety behavior at the level of 0.01.

It can be seen from the above table that the correlation coefficients between the variables were significant and passed the significance level of 1%. Moreover, the results showed that the correlation among variables was consistent with the expectation of each hypothesis.

### 4.3. Hypothesis Testing

Regression analysis is needed to further explain the causal relationship and strength between the variables [46,47]. Based on the above correlation analysis, under the control of gender and age, the relationship between variables was analyzed. According to the hypothesis, in this paper, the variables were analyzed by multiple linear regression analysis. The results are shown in Table 6.

It can be seen from model 1 to model 4 that the psychological contract has a significant negative impact on job burnout, and its influence coefficient (i.e., regression coefficient) is −0.561 (*p* < 0.01), so H3 has been verified and the psychological contract has a significant positive impact on perceived insider status, with the influence coefficient being 0.733 (*p* < 0.01), so H6 has been tested.

From the above analysis, it can be seen from model 6 that psychological contract has a significant positive impact on safety behavior, and the influence coefficient is 0.629 (*p* < 0.01); therefore, H1 has been verified. Job burnout has a significant negative impact on safety behavior in model 7, and the influence coefficient is −0.655 (*p* < 0.01), so H4 was verified. The influence coefficient of psychological contract and job burnout on safety performance is 0.436 and −0.344 (*p* < 0.01) in model 8, and the effects are significant; that is, job burnout plays a partial mediating role between psychological contract and safety behavior when employees consider the spread of COVID-19 in work resumption, and H2 has been verified. Similarly, according to the results of model 9 and model 10, it can be concluded that perceived insider status also plays a partial mediating role between psychological contract and safety behavior when employees consider the spread of COVID-19; meanwhile, H5 and H7 were verified.

Multiple linear regression verified the hypotheses of research. In order to explore the mediating effect of job burnout and perceived insider status, the model was further verified. The total effect value of the model is 0.6292 (*p* < 0.01), and the total indirect effect value is 0.2394, and the mediators’ results of indirect effects are shown in Table 7. Indirect effect 1 refers to job burnout, indirect effect 2 refers to perceived insider status, and indirect effect 3 refers to the difference between job burnout and perceived insider status.

Finally, the path analysis of the model was performed. The results show that χ^2^/df = 41.022, GFI = 0.96, RMSEA = 0.316, CFI = 0.941 and RMR = 0.018; these values indicate that the model fitting effect is good.

**Figure 2:**
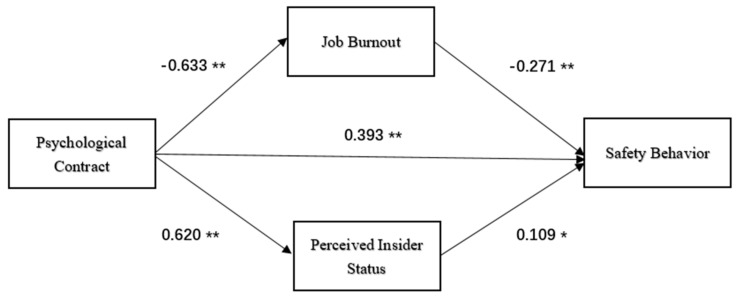
Double mediating effect. N = 402, * is significantly correlated at 0.05 level (bilateral), ** is significantly correlated at 0.01 level (bilateral).

Combined with the above tests, the following double mediating effect diagram can be obtained, and the path coefficient and significance were indicated. ** is significantly correlated at the 0.01 level (bilateral), * is significantly correlated at the 0.05 level (bilateral). The details are shown in Figure 2.

### 4.4. Summary

According to the data of regression analysis, the fitting effect is good. From the results, we can see that psychological contract has a positive predictive effect on employees’ safety behavior, and the mediating role of job burnout and perceived insider status is significant. Therefore, we can say that the psychological contract, as an important factor affecting the safety behavior of employees, has played a positive role in work resumption and ensured the safety of employees in the epidemic environment in China, which is conducive to controlling the outbreak of COVID-19. However, at the same time, enterprises should also pay attention to the mediating role of job burnout and perceived insider status, reduce the risk of epidemic situation caused by job burnout in the process of work resumption and try to improve the employees’ perceived insider status to avoid the harm of COVID-19.

## 5. Discussion

Based on the prevention of COVID-19 and work resumption of enterprises in China in 2020, this study constructs a double mediation model based on job burnout and perceived insider status as parallel mediators. The results are helpful to deepen the research on the relationship between the psychological contract and employees’ safety behavior and provide guidance for employees to strengthen the prevention of COVID-19 in work resumption as well as the establishment of a public health emergency response mechanism in a future crisis.

### 5.1. The Effect of Psychological Contract on Safety Behavior

Based on the agreement between the organization and employees in social relations or work environment, the study evaluated the impact of psychological contract on employees’ safety behavior, and discussed whether these factors will affect employees’ safety behavior. Previous studies have shown that the psychological contract can regulate the behavior of employees in their work. In the case of employees who have reached a high psychological contract with the organization, they will abide by the organizational norms strictly and reduce illegal operations, which is conducive to ensuring workers’ safety [48]. This study also verified the previous conclusion; that is, psychological contract has a positive predictive effect on employees’ safety behavior to resist the spread of COVID-19. Therefore, employees with higher psychological contract will show more safety behaviors to ensure their own safety.

### 5.2. The Role of the Mediators

To explore the mediating role of job burnout in the relationship between psychological contract and employee safety behavior, it is not only helpful to reveal which factors in the psychological contract affect employees’ safety behavior from the perspective of effort–reward but also help us to reveal the mechanism of employees’ unsafe behavior from the perspective of individual level. This study found that, in the process of coping with COVID-19, the psychological contract can predict employees’ safety behavior through the mediating role of job burnout, which shows the universality of previous studies. The negative predictive effect of job burnout on the safety behavior of employees to prevent the spread of COVID-19 is significant in the Chinese epidemic prevention environment, which is consistent with the research of most scholars; that is, the reduction of job burnout will improve the safety behavior of employees [49].

To explore the mediating role of perceived insider status in the relationship between psychological contract and employees’ safety behavior is to reveal how the psychological contract affects employees’ safety behavior from the perspective of employees’ sense of belonging, which is helpful for us to explore the mechanism of unsafe behavior at both organizational and individual levels. Previous studies have found that the psychological contract can improve employees’ perception of insider identity and organizational citizenship behavior [50], and perceived insider status can help to improve employees’ safety behavior to a certain extent [51]. This study found that, in the process of preventing the spread of COVID-19 among Chinese employees, psychological contract can predict employees’ safety behavior through the mediating role of perceived insider status; that is, individuals with high insider perception consciously control their own behavior to avoid the threat of COVID-19. This study also shows that the improvement of perceived insider status will improve employees’ conscious prevention behavior with regard to COVID-19.

### 5.3. Research Significance and Enlightenment

In this paper, we innovatively use the double mediation model to study the safety behavior of employees in the context of China’s epidemic situation in response to COVID-19. Research on mediating variables shows that employees’ conscious participation in safety precautions can better prevent the spread of COVID-19. This study not only expands the research on the related concepts of psychological contract and safety behavior but also complements the research on the mediating effect of the two concepts and provides a theoretical basis for enterprises to protect the safety of employees in an epidemic situation.

In addition, this study takes the prevention of COVID-19 in Chinese enterprises as the background, discusses the safety behavior of enterprise employees and modifies the safety behavior scale for a COVID-19 context. The modified safety behavior scale provides a research tool for exploring the safety behavior of employees to prevent the spread of COVID-19 and provides a reference for the application of the safety behavior scale in different situations. In addition, the research on the safety behavior of employees in the Chinese epidemic environment also provides some action guidance for other countries suffering from COVID-19 and difficulties in work resumption. First of all, enterprises should strengthen the popularization of epidemic prevention knowledge and safety protection, guide employees to take epidemic prevention as a part of their daily work and reduce their sense of panic about COVID-19. Secondly, enterprises should provide necessary emotional care and welfare support and improve employees’ responsibility and sense of belonging, so that they can voluntarily contribute to their work. At the same time, enterprises should activate the organizational atmosphere through group competition or communication and other collective activities, pay attention to the combination of work and rest, reduce the possibility of unsafe behavior caused by job burnout and reduce the adverse impact of the epidemic.

### 5.4. Limitations

There are still some deficiencies in this study which need to be improved in future research. First of all, this study used a questionnaire survey method to collect data in China. Due to the limited geographical and enterprise types, the research object is too concentrated, which makes the research results not necessarily universal. Because of the differences in culture and management styles between the east and the west, employees’ acceptance of the regulations for prevention of COVID-19 varies greatly, which will lead to differences in whether the psychological contract is reached or not and affect the strength of the sense of fatigue and pressure at work. For example, in some eastern countries, such as China, clear and strict regulations make employees feel secure. However, in western countries, such as the United States, the same standard may mean a lack of freedom for employees. In the future, more enterprises in various regions could be examined to verify the universality of the hypothesis. Secondly, this study mainly discusses safety behavior from the perspective of employees, lacking organizational factors. Therefore, researchers could analyze the safety behavior of employees from the perspective of the organization or the combination of organization and individual in the future and could also consider the influence of some demographic variables on the relationship among variables. Thirdly, the improvement of the safety behavior scale is based on the specific situation of the Chinese response to COVID-19, which has certain limitations and may need to be further improved and tested in the future. Finally, the dual mediation model used in this paper only tests the role of job burnout and perceived insider status and does not delve into whether there are other mediators or moderators; future research could explore and improve the theoretical model from more aspects.

## 6. Conclusions

This study shows that the psychological contract plays a positive role in preventing COVID-19 in work resumption, and the mediating role of job burnout and perceived insider status on the impact of psychological contract on employee safety behavior also plays an important role in preventing and controlling the spread of COVID-19. The psychological contract can weaken or strengthen employees’ safety behavior through job burnout or perceived insider status.

The psychological contract can promote employees to have a sense of belonging and organizational identity, reach a high degree of agreement with the enterprise and may encourage them regard themselves as internal members of the enterprise; that is to say, they will form the sense of perceived insider status and thus show a higher sense of responsibility in their work, so as to regulate their own and colleagues’ behavior to strengthen the prevention of COVID-19. In addition, the sense of belonging brought about by the psychological contract can also make employees more integrated into the organization, which helps to create a harmonious working atmosphere. It can reduce the employees’ sense of pressure and fatigue—that is, reducing the possibility of job burnout, so as to avoid the negligence of prevention of COVID-19.

These conclusions should be explored in depth and considered for awareness-raising during pandemics or other crisis situations, as they could enrich prevention interventions in public health and, in particular, in the mental health of employees. The information provided by this research is helpful for enterprises to design a program for work resumption in an epidemic situation.

## Figures and Tables

**Table 1:** The COVID-19 safety behavior scale.

Number	Item
1	I put the epidemic prevention supplies well in my work.
2	I wear the necessary anti-epidemic protective equipment as requiring.
3	I abide by the safety rules of epidemic prevention in my work.
4	I actively cooperate with the managers in the work of epidemic prevention.
5	Even if there is not any supervision, I will pay attention to epidemic prevention.
6	I (will) put forward suggestions to improve the safety of epidemic prevention.
7	I (will) take part in activities to improve the safety of epidemic prevention.
8	I (will) take the initiative to demonstrate the correct epidemic prevention methods to my colleagues.
9	I (will) take the initiative to correct the inappropriate behavior of my colleagues.

**Table 2:** Basic statistics of employees.

Characteristics	Classification	Number	Proportion (%)
Gender	Male	174	43.3
	Female	228	56.7
	Missing Value	0	0.0
Age	20 Years Old and Below	10	2.5
	21–30 Years Old	150	37.3
	31–40 Years Old	139	34.6
	41–50 Years Old	92	22.9
	51 Years Old and Above	11	2.7
	Missing Value	0	0.0
Level of Education	High School and Below	274	68.2
	Junior College	69	17.2
	Undergraduate	55	13.7
	Master’s Degree and Above	4	1.0
	Missing Value	0	0.0
Position	Staff	290	72.1
	Manager	111	27.6
	Missing Value	1	0.2
Working Years	Within 1 Year	51	12.7
	1–5 Years	188	46.8
	6–10 Years	106	26.4
	More than 10 Years	55	13.7
	Missing Value	2	0.5

**Table 3:** Factor load matrix after rotation: the COVID-19 safety behavior scale.

Dimensions	Items	Factor Load after Rotation		Variance Interpretation Rate
		Factor 1	Factor 2	
Safety Obedience Behavior	3	0.841	0.285	38.464%
	4	0.801	0.289	
	5	0.784	0.334	
	2	0.765	0.366	
	1	0.635	0.483	
Safety Participation Behavior	8	0.266	0.889	32.681%
	9	0.290	0.828	
	7	0.411	0.680	
	6	0.433	0.600	
Cumulative Variance Interpretation Rate				71.145%

**Table 4:** Results of confirmatory factor analysis.

Fit Indices	*p*	χ^2^/df	GFI	RMSEA	RMR	CFI	NFI	NNFI
Criterion	>0.05	<3	>0.9	<0.10	<0.05	>0.9	>0.9	>0.9
Psychological Contract	0.000	2.621	0.953	0.063	0.018	0.969	0.951	0.959
Job Burnout	0.000	1.854	0.954	0.046	0.023	0.966	0.930	0.958
Perceived Insider Status	0.049	2.381	0.990	0.059	0.008	0.996	0.993	0.990
Safety Behavior	0.000	5.229	0.930	0.103	0.021	0.952	0.941	0.933

N = 402, GFI is goodness of fit index; RMSEA is root mean square error of approximation; RMR is root mean square residual; CFI is comparative fit index; NFI is normalize fit index; NNFI is non-normed fit index.

**Table 5:** The results of correlation analysis.

Variable	Psychological Contract	Job Burnout	Perceived Insider Status	Safety Behavior
Psychological Contract	1			
Job Burnout	−0.633 **	1		
Perceived Insider Status	0.620 **	−0.582 **	1	
Safety Behavior	0.629 **	−0.580 **	0.508 **	1

N = 402, ** is significantly correlated at 0.01 level (bilateral).

**Table 6:** Multiple linear regression analysis results of the variables.

Outcome Variable	Job Burnout		Perceived Insider Status		Safety Behavior					
	Model 1	Model 2	Model 3	Model 4	Model 5	Model 6	Model 7	Model 8	Model 9	Model 10
Gender	0.010	0.012	−0.032	−0.046	0.038	0.026	0.039	0.030	0.052	0.033
Age	−0.140	−0.004	0.021	0.009	−0.006	−0.016	−0.015	−0.018	−0.015	−0.017
Psychological Contract		−0.561 **		0.733 **		0.629 **		0.436 **		0.509 **
Job Burnout							−0.655 **	−0.344 **		
Perceived Insider Status									0.432 **	0.164 **
R^2^	0.001	0.401	0.001	0.386	0.001	0.397	0.339	0.453	0.261	0.420
ΔR^2^	−0.004	0.396	−0.004	0.381	−0.004	0.392	0.334	0.447	0.255	0.414
F	0.140	88.772 **	0.290	83.430 **	0.267	87.306 **	67.909 **	82.054 **	46.745 *	71.825 **

N = 402, * is significantly correlated at 0.05 level (bilateral), ** is significantly correlated at 0.01 level (bilateral).

**Table 7:** Mediating effects of job burnout and perceived insider status.

Effect	Effect Value	Boot S.E.	Boot LLCI	Boot ULCI	Proportion
Total	0.2394	0.0512	0.1395	0.3404	38.05%
Indirect effect 1	0.1709	0.0436	0.0872	0.2579	27.16%
Indirect effect 2	0.0685	0.0321	0.0063	0.1310	10.89%
Indirect effect 3	0.1025	0.0570	−0.065	0.2183	16.29%

N = 402, S.E. is standard error; LLCI is lower level of confidence interval; ULCI is upper level of confidence interval.
